# Morphological, Gene, and Hormonal Changes in Gonads and In-Creased Micrococcal Nuclease Accessibility of Sperm Chromatin Induced by Mercury

**DOI:** 10.3390/biom12010087

**Published:** 2022-01-06

**Authors:** Gennaro Lettieri, Nadia Carusone, Rosaria Notariale, Marina Prisco, Alessia Ambrosino, Shana Perrella, Caterina Manna, Marina Piscopo

**Affiliations:** 1 Department of Biology, University of Naples Federico II, Via Cinthia, 21, 80126 Naples, Italy; gennarole@outlook.com (G.L.); nadia.carusone@libero.it (N.C.); marina.prisco@unina.it (M.P.); alessia.ambrosino@gmail.com (A.A.); shana.perrella@yahoo.it (S.P.); 2 Department of Precision Medicine, School of Medicine, University of Campania “Luigi Vanvitelli”, Via Luigi de Crecchio, 80138 Naples, Italy; notarialer@gmail.com (R.N.); caterina.manna@unicampania.it (C.M.)

**Keywords:** mercury, *Mytilus galloprovincialis*, protamine-like proteins genes, gonad, spermatozoa, micrococcal nuclease digestion, hormones, morphology

## Abstract

Mercury is one of the most dangerous environmental pollutants. In this work, we analysed the effects of exposure of *Mytilus galloprovincialis* to 1, 10 and 100 pM HgCl_2_ for 24 h on the gonadal morphology and on the expression level of three stress genes: *mt10*, *hsp70* and *πgst*. In this tissue we also evaluated the level of steroidogenic enzymes 3β-HSD and 17β-HSD and the expression of PL protein genes. Finally, we determined difference in sperm chromatin accessibility to micrococcal nuclease. We found alterations in gonadal morphology especially after exposure to 10 and 100 pM HgCl_2_ and hypo-expression of the three stress genes, particularly for *hsp70*. Furthermore, decreased labelling with both 3β-HSD and 17β-HSD antibodies was observed following exposure to 1 and 10 pM HgCl_2_ and complete absence at 100 pM HgCl_2_ exposure. Gonads of mussels exposed to all HgCl_2_ doses showed decreased expression of PL protein genes especially for PLIII. Finally, micrococcal nuclease digestions showed that all doses of HgCl_2_ exposure resulted in increased sperm chromatin accessibility to this enzyme, indicative of improper sperm chromatin structure. All of these changes provide preliminary data of the potential toxicity of mercury on the reproductive health of this mussel.

## 1. Introduction

The increasing worldwide levels of pollutants in the marine environment require control and monitoring strategies. Mercury is an extremely toxic heavy metal in the marine environment and its effects on marine organisms are complex and often unpredictable. The United Nations Environment Programme (UNEP)’s “Global Mercury Assessment” estimated that anthropogenic activities have increased current atmospheric mercury concentrations by approximately 450% above natural levels, resulting in a substantial increase in seawater [1]. In marine environments, mercury is subject to bioaccumulation and biomagnification, and its seawater contamination is posing a serious health problem for humans [2,3] and marine organisms [4,5].

In environmental biomonitoring programs, the use of bioindicator organisms represents one of the most suitable methods for the assessment of trace metal toxicity [6]. Bivalves are excellent organisms as bioindicators of heavy metal pollution due to their characteristics including worldwide distribution, sedentary filter-feeding, easy sampling and reasonable size [7].

*Mytilus galloprovincialis* (*M. galloprovincialis*) is a readily available organism and widely distributed in the Mediterranean. This bivalve mollusc accumulates contaminants in its tissues mainly in the hepatopancreas, kidney, gills and gonads [8]. This organism responds quickly and effectively to the presence of many environmental pollutants, including heavy metals [9]. Exposure of *M. galloprovincialis* to various heavy metals causes alterations in its reproductive system [9,10,11,12,13,14]. In the sperm chromatin of *M. galloprovincialis* the main basic protein component is represented by the Protamine-like (PL) proteins [15]. In previous works we have found that the exposure to subtoxic doses of copper causes an increase in the binding affinity of PL-proteins to DNA and alterations in the expression of spermatozoa *mt10* gene [12]. Finally, alterations, such as a reduced DNA binding affinity of PL-proteins [9], and an increased *mt20* gene expression [14] following exposure to subtoxic doses of cadmium, have been reported. *Mytilus* spp. have shown a greater capacity for mercury (Hg) accumulation than other bivalve species [16], and recently, we evaluated the effects of exposure to mercury doses (1, 10 and 100 pM HgCl_2_), similar to those found in the Mediterranean basin and North Atlantic oceans, on *M. galloprovincialis* male reproductive system [17]. In particular, we showed bioaccumulation of mercury in the male gonads and alterations in the expression of spermatozoa *mt10* and *hsp70* stress genes. We also found several changes in the PL-proteins, such as a reduced ability to bind DNA and an inability to protect DNA from oxidative damage [9]. Furthermore, at these doses of HgCl_2_ exposure, the formation of PL protein aggregates and alterations in their structural conformation was shown to result in changes in both their binding to DNA and their release from sperm nuclei [10].

In order to obtain further useful information on the effects of these HgCl_2_ exposure doses on the male reproductive system of *M. galloprovincialis*, in the present work we evaluated possible morphological changes in the gonads by hematoxylin and eosin (H&E) staining and the levels of steroidogenic enzymes 3β-HSD and 17β-HSD by immunohistochemistry. In addition, by quantitative reverse transcription polymerase chain reaction (RT-qPCR), gene expression of *mt10*, *hsp70*, *πgst*, and genes encoding PL proteins were also measured in the gonads. Finally, the possible difference in sperm chromatin accessibility to micrococcal nuclease (MNase) in mussels unexposed and exposed to these doses of mercuric chloride was investigated.

## 2. Materials and Methods

### 2.1. Ethics Statement

This research was performed on the marine invertebrate *M. galloprovincialis* (Lamarck, 1819), which is not protected by any environmental agency in Italy. This study was conducted in strict accordance with European (Directive 2010/63) and Italian (Legislative Decree n. 116/1992) legislation on the care and use of animals for scientific purposes.

### 2.2. Mussels Sampling and Exposure to HgCl_2_

Adult mussels of *M. galloprovincialis*, medium size of the shell length 4.93 ± 0.17 cm, were supplied by Eurofish Napoli S.R.L. Baia, in Naples and used in this study. Fifteen mussels of unknown sex were exposed to 1, 10 and 100 pM HgCl_2_, as previously described for the other heavy metals [18] in laboratory plastic tanks (36 cm × 22 cm × 22 cm) containing 6 L of 33‰ artificial sea water (ASW) with the following composition for 1 L: NaCl 29.2 g, KCl 0.60 g, MgCl_2_ 1.2 g, NaHCO_3_ 0.20 g and CaCl_2_ 1.08 g. In each tank, mussels were exposed to a single dose of HgCl_2_. The exposure was performed at 18  ±  1 °C, for 24 h changing water and metal salts every 12 h during treatment and recording dissolved oxygen and temperature at predetermined time intervals. The experiments were conducted in January of the current year. Tanks containing only ASW were used as a control for nonexposed mussels.

### 2.3. Gonad Sampling and PL Proteins Extraction

After 24 h of exposure to the three HgCl_2_ doses, gonads were collected from male specimens. Mussels were opened forcing the valves with a knife, being care not to cut soft tissues. Mussels’ sex was initially identified observing the colour of mantle and confirmed by light microscope observation of gonads smear. Briefly, the piece of gonad was smeared on the microscope slide and as soon as it was dry, it was observed under the light microscope. Each identified gonad was stocked and stored at −80 °C.

Sperm-filled gonads were used for acid extraction of PL proteins. Briefly, one gonad from each condition was mechanically pestled in distilled water. Subsequently, centrifugation was performed at 1000× *g* for 2 min at 4 °C to remove debris, and from the obtained supernatant, PLs were extracted by 5% perchloric acid as described in Piscopo et al. 2017 [14].

### 2.4. RNA Extraction, cDNA Synthesis and RT-qPCR

Total RNA was purified from gonads of nonexposed mussels (control) and exposed to 1, 10, 100 pM HgCl_2_ using Trizol reagent (Invitrogen, Carlsbad, CA, USA) according to the manufacturer’s instructions. The RNA obtained was quantified by a UV-Vis spectrophotometer (NanoDropH ND-1000, Waltham, MA, USA). Moreover, electrophoretic analysis was performed on 1% agarose gels under denaturing conditions to assess its quality. Genomic DNA was removed from the samples with an Ambion (Austin, TX, USA) DNA-free kit. cDNA was synthesized from 1 µg of RNA from each samples using M-MLV reverse transcriptase (ImpProm II kit, Promega, Madison, WI, USA). The RT-qPCR was carried out as described in Lettieri et al. [11]. In particular, to determine the gene expression was used 100 ng of the cDNA with 10 µM of each forward and reverse primers in a final volume of 50 µL using SYBRGreen PCR Master Mix Kit (Applied Biosystems, Foster City, CA, USA) with the 7500 Real Time PCR System (Applied Biosystems, Foster City, CA, USA). Each PCR reaction was conducted for 40 cycles as described in Lettieri et al., 2019 [19] maintaining the following conditions: Denaturation at 95 °C for 15 min; annealing and elongation at 60 °C for 1 min. The primer used for the reaction was engineered using the open-source software Primer3, following the sequences and the accession numbers reported in Table 1. The results were exported into Microsoft Excel (Redmond, WA, USA, ver. 2009—build 13231.20262) from the ViiA™-7 Software (Foster City, CA, USA). The relative quantification of gene expression was evaluated using the the ∆∆Ct method as reported in Livak et al. [20]. The expression of *mt10*, *hsp70*, *πgst*, *PL-III* and *PL-II/IV* genes was compared to those obtained from control mussels. In Table 1 was reported the list of primers used.

### 2.5. Sperm Nuclei Preparation and MNase Assay

For the preparation of sperm nuclei the procedure reported in Olivares and Ruiz 1991 [21] was followed. In brief, sperm nuclei were obtained starting from spermatozoa pellet of mussels exposed to different HgCl_2_ doses. Spermatozoa pellets were obtained centrifuging semen for 10 min at 1900× *g* at 4 °C. Each pellet was then resuspended in 1 ml of a solution (solution 1) with the following composition. -NaCl 0.15 M- EDTA pH 8 25 mM PMSF 1 mMF and the sample was centrifuged at 4 °C for 10 min at 1900× *g*. Subsequently, the obtained pellet was resuspended in the following buffer (solution 2): 0.25 M sucrose- MgCl_2_ 5 mM- Tris-HCl pH 8 10 mM- PMSF 1 mM- Triton X-100 0.38% and then centrifuged at 4 °C for 10 min at 1900× *g*. The pellet was recovered and washed twice with the previous buffer in the absence of Triton. The prepared sperm nuclei were resuspended in the following buffer (solution 3)-NaCl 0.15 M- Tris HCl 10 mM pH 8- CaCl_2_ 0.5 mM and subjected to MNase assay. Digestion was carried out with 10 units of enzyme at 37 °C for 1 mg/mL DNA concentration (A260 = 20) and the reaction stopped at different times (5′, 10′, 15′, 30′ and 60′) adding 2 mM EDTA pH 8 at ice. The digestion products were centrifuged at 1900× *g* for 10 min at 4 °C. This produced a pellet (P) and a supernatant (S). P was used for DNA extraction following a common extraction phenol/chloroform/isoamyl alcohol. Briefly, 1 M NaCl and 0.5% SDS was added to P and incubated 30 min at RT. Equal volume of phenol/chloroform/isoamyl alcohol (25:24:1) was added, samples were vortexed and centrifuged at 4 °C for 5 min at 13,000× *g*. Successively, 0.3 M NaCl and 2.5 volumes of ethanol 100% was added to the aqueous phase to precipitate the DNA at −20 °C for 1 h.

### 2.6. Morphological Analyses of Gonad

For light microscope investigations, samples 0.5 cm × 0.5 cm were taken from the central portion of one hemi-mantle. Specimens were fixed in Bouin’s solution for 24 h, dehydrated in alcohol and embedded in paraffin wax. Five µm thick sections were cut and stained with hematoxylin and eosin stain.

### 2.7. Immunohistochemistry Analyses

For immunohistochemistry, 5 µm thick sections of Bouin fixed specimen on poly-l-lysine slides were deparaffinized and rehydrated in xylene and a series of graded alcohols. The sections were treated with 10 mM citrate buffer pH 6.0 in the microwave for antigen retrieval and then incubated in 2.5% H_2_O_2_ in methanol for endogenous peroxidase blocking. Non-specific background was reduced with the incubation in 3% BSA in PBS buffer for 1 h at room temperature. Sections were then treated overnight at 4 °C with the primary rabbit antibodies diluted in 1% BSA in PBS buffer: (1) anti-human 3β-HSD (1:50), (2) anti-mouse 17β-HSD (1:50). In the first instance, antibodies specificity was tested by making an immunoblot analysis on *Mytilus galloprovincialis* gonadal proteins as previously reported by Prisco et al. (2017) [22]. The reaction was revealed with a peroxidase-conjugated goat anti-rabbit secondary antibody, using DAB (Roche) as chromogen. Negative controls were carried out by omitting primary antibodies. An immunohistochemical signal was observed using a Zeiss Axioskop microscope; images were acquired by using AxioVision 4.7 software (Zeiss).

### 2.8. Statistical Analysis

The ANOVA test followed Tukey’s post-hoc test was performed for gene expression analysis. The statistical analyses were performed by GraphPad Prism 9 (ver. 9.1.2 (226)) (GraphPad Software, San Diego, CA, USA).

## 3. Results

### 3.1. Gonadal mt10, πgst and hsp70 Expression

**Figure 1:**
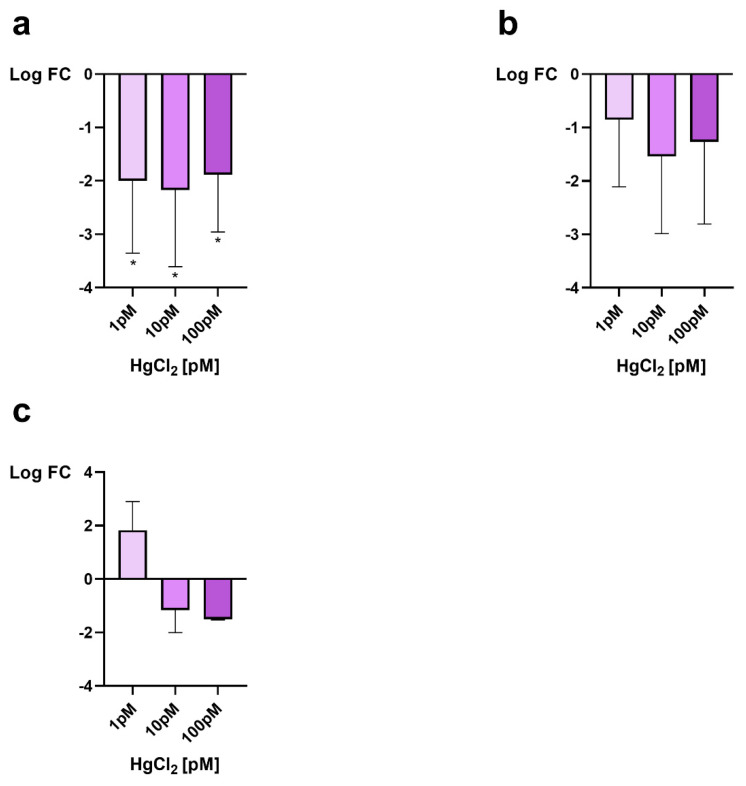
RT-qPCR expression analysis of *M. galloprovincialis* gonadal *hsp70*, *πgst* and *mt10*. In the figure, the change in expression, respect to the control condition (nonexposed mussels), of *hsp70* (**a**), *πgst* (**b**) and *mt10* (**c**) is reported under the three HgCl_2_ exposure conditions. Expression was determined with respect to the GAPDH housekeeping gene. Values are presented as mean ± S.D. (*n* = 6); asterisks indicate a statistically significant difference from unexposed mussels: * = *p* < 0.05.

The RT-qPCR was used to assess the expression of *mt10, πgst* and *hsp70* as a response to possible stress in male gonads after mussel exposure to 1, 10 and 100 pM HgCl_2_. All genes were downregulated after exposure of mussels to all doses of HgCl_2_ except for *mt10* gene after exposure to 1 pM HgCl_2_ in which an overexpression, approximately of 2-fold, of this gene, was observed (Figure 1c).

*Hsp70* gene showed a similar decrease of expression for all exposure doses, a little more pronounced after exposure to 10 pM HgCl_2_, where the reduction was of about 2.2 times compared to the control condition (Figure 1a). Downregulations were also found both for the πgst gene (Figure 1b) and for the *mt10* gene (Figure 1c) but to a lesser extent (1–1.5-fold).

### 3.2. Morphological Analisys of Gonad

**Figure 2:**
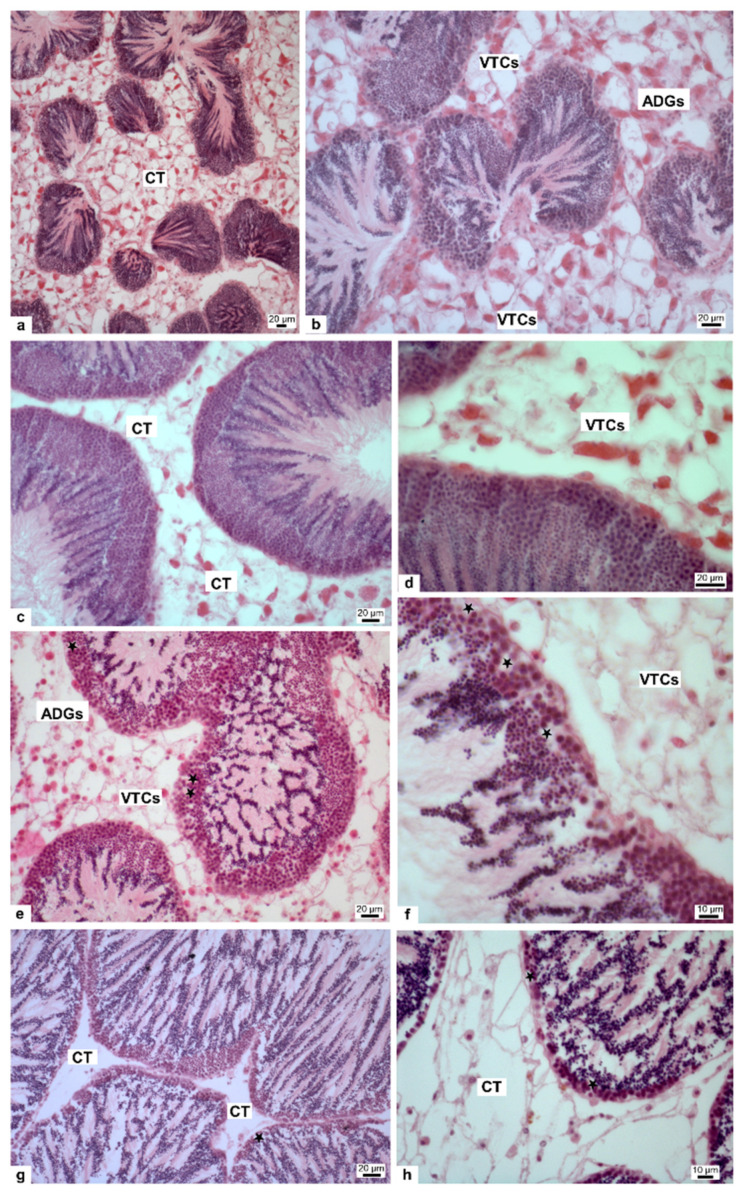
Staining of *M. galloprovincialis* gonad with hematoxylin and eosin. Gonad morphology of nonexposed (**a**,**b**) and exposed mussels to 1 (**c**,**d**), 10 (**e**,**f**) and 100 pM (**g**,**h**) HgCl_2_, respectively. CT: connective tissue; VTCs: vesicular connective tissue cells; ADGs: adipogranular cells; * (star): degenerative vacuolized areas.

Given the alteration of the two stress genes in the gonad after exposure of mussels to all HgCl_2_ doses, we evaluated possible morphological alterations in this tissue. Testicular sections of control mussel showed normal spermatogenesis and cell arrangement in the spermatic follicles and the connective tissue formed by adipogranular cells (ADGs) and vesicular connective tissue cells (VTCs) resulted normally structured (Figure 2a,b). In 1 pM HgCl_2_ treated mussels the sperm follicles resulted like the control (Figure 2c,d), whereas in 10 and 100 pM HgCl_2_ treated mussels, degenerative vacuolized areas in spermatogenic layers were visible (stars) (Figure 2e,h). Spermatogenesis appeared to proceed normally, but connective tissue (CT) showed increasing disorganization and degeneration in relation to increasing HgCl_2_ dose; ADGs were no longer distinguishable in samples treated with 10 and 100 pM HgCl_2_ (Figure 2f,h). Finally, in samples treated with 100 pM HgCl_2_ the CT appeared totally disorganized (Figure 2h).

### 3.3. Immunohistochemistry

**Figure 3:**
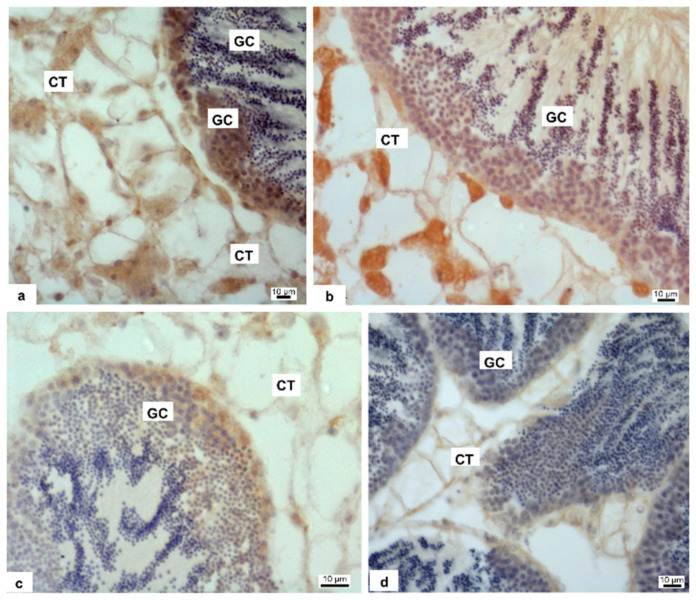
Immunolabeling of *M. galloprovincialis* male gonad with anti-3β-HSD antibody. Brown coloration indicates the enzyme presence. Nonexposed (**a**) and exposed mussels to 1 (**b**), 10 (**c**) and 100 pM (**d**) HgCl_2_, respectively. Germ cells (GC) are positive to antibody in control and 1 and 10 pM treated samples (**a**–**c**); the positivity disappears almost completely in 100 pM treated samples (**d**) In the connective cells (CT, connective tissue) the 3β-HSD enzyme is recognizable in control and 1 pM treated animals (**a**,**b**); in 10 and 100 pM treated samples the antibody positivity is poor (**c**) or totally disappeared (**d**).

Anti-3β-HSD and anti-17β-HSD antibodies showed the presence of both enzymes in the gonad, in overlapping manner, so we present in the figure the localization of 3β-HSD only. The enzymes are present in connective tissue and in germ cells (GC) of control specimens (Figure 3a). In the specimens treated with 1 pM HgCl_2_ (Figure 3b), the labelling on connective tissue (CT) was still present, decreasing instead in germ cells. Antibody positivity decreased even more in specimens treated with 10 pM HgCl_2_ (Figure 3c) and completely disappeared in specimens treated with 100 pM HgCl_2_ (Figure 3d).

### 3.4. Gonadal PL-Proteins Genes Expression

**Figure 4:**
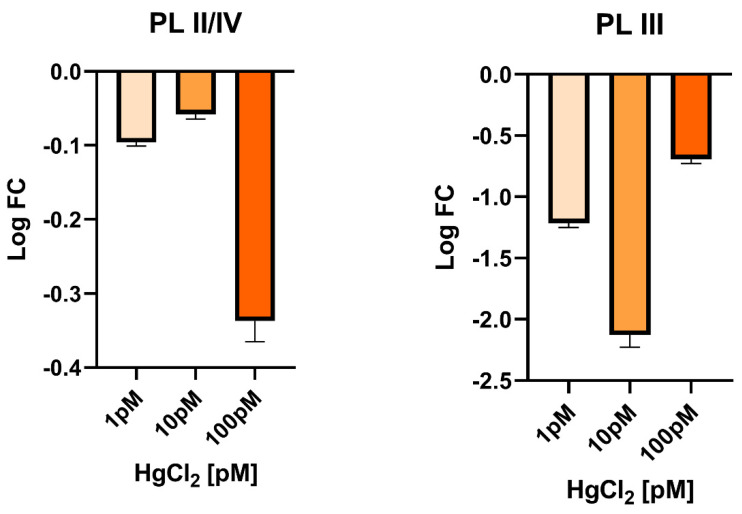
RT-qPCR expression analysis of *M. galloprovincialis* gonadal PLIII and PLII/IV genes. In the figure, the change in gene expression is reported under the three HgCl_2_ exposure conditions compared to the control condition (nonexposed mussels: CTR). Expression was determined with respect to the GAPDH housekeeping gene. Values are presented as mean ± S.D. (*n* = 6).

Considering the morphological changes and enzymes changes involved in hormones synthesis found in the gonad of mussels exposed to these doses of HgCl_2_, we also evaluated in this tissue the expression levels of genes encoding PL-proteins, as these are the major basic protein component of the sperm chromatin of this organism. In gonads, the *PLII/IV* was found to be slightly downregulated under all exposure conditions. A more marked downregulation was instead detected for the PL-III gene, under all exposure conditions but particularly at 10 pM HgCl_2_, where an approximately 2-fold decrease was measured (Figure 4).

### 3.5. MNase Digestion Pattern of M. Galloprovincialis’sperm Nuclei

**Figure 5:**
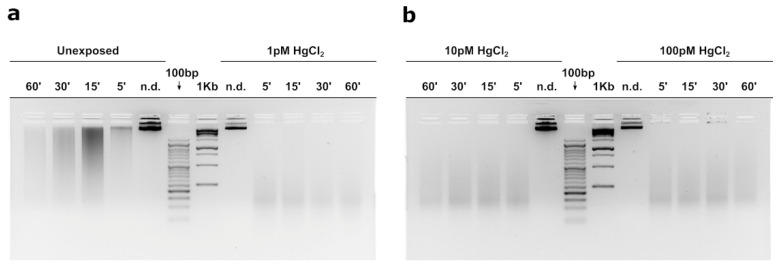
Micrococcal nuclease (MNase) digestion time course of *Mytilus galloprovincialis*’ sperm chromatin. Analyses of DNA by electrophoresis on agarose gel: unexposed and exposed mussels to 1 pM HgCl_2_ (Panel (**a**)), exposed mussels to 10 and 100 pM HgCl_2_ (Panel (**b**)). n.d. = undigested sperm genomic DNA. 1Kb and 100 bp: molecular weight markers.

Figure 5 shows the MNase digestion time course of *M. galloprovincialis’* sperm chromatin for 5, 15, 30, and 60 min. The analyses were conducted on sperm chromatin of mussels nonexposed and exposed to the three HgCl_2_ exposure doses. In this approach, DNA contents of the fractions obtained at different times of MNase digestion were analysed. The results show that in unexposed mussels (Figure 5a) MNase digestion of sperm chromatin results in the electrophoretic pattern typical of sperm chromatin of this organism. Chromatin appears mainly as a smear, which is indicative of the absence of a typical nucleosomal organization. In contrast, following exposure to all doses of HgCl_2_, the sperm chromatin DNA appears fully degraded upon digestion with Mnase as early as the lowest digestion time (5′), indicative of an improper sperm chromatin structure (Figure 5a,b).

## 4. Discussion

Mercury (Hg) is a global environmental pollutant that affects ecosystem health and marine organisms [23,24], due to its significant implications on several biological processes, including growth, sexual maturation, and reproductive success [25]. Contamination of toxic substances in the environment, such as mercury, has been known to have socio-economic consequences. These effects have led to a limitation of Hg emissions from large-scale human activities. Bivalves are important species in coastal ecosystems, and play an important role particularly in food webs (as suspension feeders) and represent a significant fraction of fishery resources. In recent years, mussels have become a commercially important seafood species worldwide. Although the consumption of mussels is considered a safe food, bioaccumulation of heavy metals remains a problem for mussel consumers and this has an impact not only on human health but also on the economy [26]. Mussels, in fact, have strong interactions with the environment, water, and sediments and are considered good bioindicator species. In particular, *M. galloprovincialis* is an important food source, widespread throughout the world with high ability to bioaccumulate pollutants. So, the ingestion of drug-contaminated bivalves can pose a health risk to the human population [27], e.g., potential antibiotic resistance [28]. Thus, many pollutants can reach humans through mussels. These include: carbamazepine (CBZ), an antiepileptic drug used in the treatment of epilepsy, neuropathic pain, and psychiatric disorders [29], microplastics [30], and heavy metals, such as mercury (Hg), lead (Pb), chromium (Cr), and cadmium (Cd). Long-term exposure to these substances can produce neurological, physiological, and physical problems [31].

Mussels have also shown a greater capacity for Hg accumulation than other bivalve species [16], and the gonads have a particular affinity for mercury like other endocrine organs [32].

For these reasons and considering that the combination of morphological changes and transcriptional responses is recognized as valuable and promising tools for the study of ecotoxicological effects [33], in the present work, we first investigated changes in male gonadal morphology and the level of steroidogenic enzymes after exposure of *M. galloprovincialis* for 24 h to 1, 10, 100 pM HgCl_2_. In addition, we assessed both the expression levels of three stress genes and the PL protein-coding genes and determined the difference in the accessibility of sperm chromatin to micrococcal nuclease at these doses of exposure to HgCl_2_.

First of all, we registered gonadal stress because RT-qPCR analyses showed altered expression of the stress genes, *mt10*, *π-gst* and *hsp70*. As reported by several authors [34,35] Mytilus species are able to synthesize metallothionenins, encoded by mt genes, in order to eliminate accumulated metals. Metallothionenins (MTs) are expressed in tissue, cell, and isoform specific modes depending on the type of metal they are exposed, including essential metals like copper and zinc and nonessential metals like cadmium and mercury. Banni M. et al., 2007 [36], based on laboratory exposures indicated that copper, zinc, mercury and cadmium, are able to induce the expression of this stress gene in mussels. Literature has reported work indicating alterations in *M. galloprovincialis mt10* gene expression after accumulation of various toxic metals, both essential, such as copper and zinc, and non-essential, such as cadmium and mercury but prevalently in digestive gland [37] in which, generally an increase of expression of these genes has been observed. However, there is a distinction between zinc and copper because these two metals act differently. First of all, Zn is a DNA stabilizer involved in transcription. It does not generate free radicals unlike Cu which is necessary for some redox reactions. Moreover, it can generate like Fe some free radicals chain reactions. Zn can displace Hg from interacting with proteins. In fact, it is sometimes administered in mammals as an anti Hg poisoning. Moreover, Hg uptake is decreased in the presence of Zn and recently have been reported new insight on the genetic basis of Zn and Cu accumulation in molluscs [38,39]. Zinc is an essential trace element for spermatogenesis. In mammals the Cu/Zn ratio is a marker of oxidative stress and several studies have suggested that later germ cells are less tolerant to ROS than early-type germ cells, mainly because of their limited reserve of antioxidant enzymes. This is due to the fact that zinc levels decrease during spermatogenesis. Zinc, in fact, plays an important role as a DNA stabilizer, being essential for several DNA repair enzymes that are important during early embryogenesis [40] and modulates SOD activity [41].

In the gonad of *M. galloprovincialis*, increased gene expression of *mt20* and *hsp70* was reported after exposure to cadmium in [13,14] whereas in our present study, we observed a decrease in the expression of both *mt10* and *hsp70* after mercury exposure, consistent with what was achieved after exposure of *M. galloprovincialis* to copper [12].Also π-gst genes was hypo-expressed after exposure of mussels to all doses of HgCl_2_.

In our present study, after 1 pM HgCl_2_ exposure, there was an approximately 2-fold increase in expression of *mt10* gene, while after 10 and 100 pM HgCl_2_ exposure, we observed a hypo-expression of this gene which resulted of 1.2- and 1.5-fold, respectively, compared to the control condition. To our knowledge, there is no direct correlation between *mt10*, 3β-HSD and 17β-HSD expression. A possible relationship, at the transcriptional level, between these genes has never been reported in literature. However, it cannot be excluded that mercury, at this dose, may similarly affect the transcription factors regulating the expression of these genes. A hypoexpression was also observed for *mt20* gene in mussel digestive gland after exposure to copper (Cu) [36]. In spermatozoa, however, as demonstrated in Piscopo et al., 2021 [11], *mt10* gene did not show significant differences after 1 pM HgCl_2_ exposure, while an increase in expression levels after mussels exposure to 10 and 100 pM HgCl_2_ was found, in particular an increase of about three times in the expression of this gene.

The different response found in mussels spermatozoa and gonads exposed to HgCl_2_ could suggest higher effectiveness of spermatozoa in the response to mercury, compared with gonads. A different response from spermatozoa and gonads was previously observed in response to subtoxic concentrations of copper [10].

Heat shock proteins (HSPs) are a group of ubiquitous proteins witch expression is activated by thermal stress [42]. Other stimuli that influence cell protein structure and function, including oxidative stress and heavy metals, can induce heat shock genes [43]. In unstressed cells, some members of the HSP family are produced constitutively and operate as molecular chaperones, assisting protein maturation steps such as folding, unfolding, and translocation across membranes [44]. HSPs are classified based on their molecular weight. The stress-inducible *hsp70* and constitutively expressed hsc70 proteins are well-known members of the HSP70 (70 kDa) multigene family. HSP70 acts as a cell damage defence system, whereas HSC70 acts as a molecular chaperone. In our present research stress-inducible *hsp70* gene showed a hypo-expression in all exposure doses, more pronounced at 10 pM HgCl_2_ exposure condition and in the order of approximately 2.2-fold compared to the control condition. A hypo-expression of *hsp70* gene was also find in *M. galloprovincialis* spermatozoa after 1 and 10 pM HgCl_2_ exposure [11]. To our knowledge, this is the first time that has been demonstrated a downregulation of the *hsp70* gene in gonads under metallic stress at low concentrations and within short acute exposure, as well as in spermatozoa [11]. However, this is not the first time that downregulation of this gene has been observed in response to a xenobiotic. In fact, a decrease in the expression of *hsp70* gene has been observed in response to paracetamol in blue mussel *Mytilus edulis* [45]. *Hsp70* preserves protein integrity and suppresses apoptosis directly, whereas thermal and oxidative stress damage protein structure. *Hsp70* is implicated in inducible stress and is involved in numerous biological processes: cancer, autophagy [46], apoptosis [47] and necrosis [48]. This implies that when HSP70 is downregulated in mussels exposed to this elements, a greater risk of apoptosis and cell death is induced [45].

Hypo-expression was also found for the *π-gst* gene, the main glutathione s- transferase isoform expressed in mussel tissues [49]. Also in this case, no linearity was observed with respect to the three exposure doses. The most marked hypo-expression was obtained after 10 pM HgCl_2_ exposure, and was of the order of 1.5-fold compared to control condition. One possible explanation for down-regulation of *π-gst* gene could reside in a potent activation of the lysosomal-vacuolar system and, subsequently, in the enhancement of autophagy [50], a condition that may mask anabolic processes such as increased gene transcription, in agreement with the hypothesis of Dondero et al., 2006 [51].

The gonadal stress responses observed by RT-qPCR prompted us to investigate on the possible damages mercury-induced on this tissue. We found that the treatment with 1 pM HgCl_2_ did not give morphological changes in the gonad respect to the control, while the two other doses, 10 and 100 pM HgCl_2_, caused vacuolized areas between the germ cells and the complete disorganization of the connective tissue. We used gonads at the mature stage, on mussels obtained in January and vacuolized areas were observed only in mussels exposed to HgCl_2_ and not in control condition. The observed morphological changes of seminiferous follicles, as vacuolization and decrease of germ cell compartment thickness, were reported also in fish [52] and in mammal testis [53,54] after HgCl_2_ treatment. A growing number of studies show that sex steroids are widespread in molluscs [55]. Initially, they were thought to be taken through the diet, since many plant species contain sex steroids similar to those in vertebrates [56]. Nevertheless, numerous studies have shown that the major classes of molluscs, i.e., cephalopods, gastropods, and bivalves, are capable of synthesizing sex steroids from precursors such as cholesterol or pregnenolone [57,58,59]. Actually, most of the steroidogenic pathways described for vertebrates have been shown to occur in molluscs.

In literature, it has been already demonstrated the negative impact of mercury on spermatogenesis and also steroidogenesis: in male catfish exposed to organic and inorganic mercury the activity of 3β-HSD was inhibited completely [60], as we observed in Mytilus treated with the higher HgCl_2_ doses. 3β-HSD is implicated in the conversion of dehydroepiandrosterone (DHEA) in androstenedione. The 3-HSD family of enzymes also catalyzes the synthesis and/or degradation of 5-androstanes and 5-pregnanes. As a result, 3β -HSD regulates key steroid hormone-related responses in the adrenal cortex, gonads, placenta, liver, and other peripheral target organs. In general, all steroid hormones, including glucocorticoids, mineralocorticoids, progesterone, androgens, and oestrogens, require 3β-HSD for production [61]. 17β-HSD, on the other hand, mediates the conversion of androstenedione (ASD) to testosterone (T). However, 17β-HSD have a wide range of functions, such as the regulation of not only steroids but also fatty acid and bile concentrations [62]. Our results showed a positive reaction after labelling with both anti-3β-HSD and anti-17β-HSD antibodies at the level of germ cells and connective tissue in the control specimen as previously reported [22], germ cells are positive to antibody in 1 pM and 10 pM treated samples also, but the positivity disappears almost completely in 100 pM treated samples. In the connective tissue the steroidogenic enzymes are recognizable in control and 1 pM treated animal, whereas in 10 and 100 pM treated samples the antibody positivity is very poor. A decrease in spermatogenesis, 17β-HSD, 3β-HSD and also testosterone level in plasma was shown in male mice after exposure to arsenic trioxide [32]. In the light of these results, we examined on gonads the expression of genes encoding PL-proteins, as these are the major component of the basic proteins that constitute the sperm chromatin of this organism [63]. A decrease of expression was found in the case of the PLIII gene in exposed mussels. The downregulation was observed at all exposure conditions but in particular after exposure to 10 pM HgCl_2_. In addition, a slight hypo-expression of PLII-PLIV was detected. These results highlights the negative impact of mercury also on gonadal protamine-like (PL) genes, proteins essential for correct DNA compaction in *M. galloprovincialis* sperm heads. A similar result was found after mussels exposure to sub-toxic concentrations of copper [12]. These alterations in the PL gene could be associated with changes in 3β-HSD and 17β-HSD expression that we observed by immunolabelling as mercury has already been shown to disrupt hormones, including testosterone [64]. Both enzymes play an important role in hormone regulation, in particular for testosterone production and its involvement in spermatogenesis [22]. These results are in line with those observed in experiments previously conducted following exposure to sub-toxic copper concentrations [12] and those relating to the accumulation of Polycyclic Aromatic Hydrocarbons (PAH) and heavy metals in the male gonad of *M. galloprovincialis* showing that these pollutants disrupts spermatogenesis and produce alterations in somatic and germ cells [65]. The testicular toxicities of mercurials, including impaired spermatogenesis and/or steroidogenesis, have been demonstrated in a number of laboratory animal species: fish [50,66,67,68,69], fowls [67]. Furthermore, this has also been demonstrated in the mouse [66,70].

The morphological and hormonal alterations observed in the gonad and the changes found for both stress genes and those encoding PLs prompted us to investigate possible alterations in the sperm chromatin structure of mussels exposed to the three doses of HgCl_2_. Results obtained by digestion with MNase show substantial differences between control and treated at all three doses of HgCl_2_. In fact, the DNA of the sperm nuclei was totally hydrolyzed after digestion with MNase in mussels exposed to all exposure conditions, contrary to what was observed in unexposed mussels in which, despite there is no nucleosomal organization, and then a smear is obtained after sperm chromatin digestion of spermatozoa with MNase but not a total degradation of DNA. These results are in line with the alterations we had highlighted in our previous works showing a difference in PL conformation upon exposure of mussels to HgCl_2_ and their different binding to DNA [10,11]. The release of PL proteins from sperm nuclei was also altered especially for PLII and PLIII at all doses of HgCl_2_ exposure [10]. Far more significant was the evidence, we have already provided, concerning the effects of the addition of PL from mussels exposed to all three HgCl_2_ doses to a plasmid DNA under pro-oxidant conditions. The result showed the breakage of DNA and a non-protection of the latter unlike what happened with PL from mussels not exposed [10,71,72]. Thus, the result we obtained with MNase digestion are quite predictable as it indicates that in mussels exposed to these doses of HgCl_2_ there might be an improper structure of the sperm chromatin. This could indicate a possible risk in the fertilizing capacity of spermatozoa from mussels exposed to these doses of HgCl_2_. After all, in literature, mercury is recognized as a male reproductive toxicant. In fact, studies performed in vitro have shown that this metal induces DNA breakage in spermatozoa and causes a decrease in sperm motility. Our previous work provides insights into the mechanisms of mercury toxicity on the reproductive system of *M. galloprovincialis*. Specifically, the alteration of PL-proteins binding to DNA induced by exposure to these HgCl_2_ doses [10]. This is in line with the results obtained with MNase digestion and could indicate an alteration in the structure of sperm chromatin, which is known to be critical for the swimming ability of spermatozoa and their ability to fertilize. In any case, however, our results are currently only descriptive as fertilization tests with spermatozoa of mussels exposed to these doses of HgCl_2_ have not yet been conducted. Therefore, it cannot be predicted at this time whether this may have a negative impact from an economic point of view. Further study will allow to understand if the genotoxic effect of HgCl_2_ in mussels could become an environmental problem since our ultimate goal is to develop genotoxicity tests based on sperm chromatin status.

In conclusion, the data obtained in this work could be an important step in understanding the mechanisms of mercury toxicity and reveal responses in a tissue such as the gonad that is generally not considered for ecotoxicological studies. In addition, our results again emphasize the importance of using *M. galloprovincialis* as a sentinel organism and suggest a possible risk on the fertilizing capacity of spermatozoa in mussels exposed to these doses of HgCl_2_ as revealed by the results of experiments conducted with MNase. However, these results will need to be further investigated with additional studies to understand how these mechanisms impact on the reproductive health of *M. galloprovincialis*.

Moreover, heavy metal contamination can induce an ecological imbalance in the receiving environment and on the diversity of aquatic organisms. Pollutants accumulate in the food chain and are responsible for harmful effects on the occurrence of various diseases, such as Minamata disease (organic mercury poisoning). Therefore, preventive measures are needed to reduce the intensity of heavy metal pollution in the aquatic environment [73].

## Figures and Tables

**Table 1:** List of forward and reverse primers used for amplification of each gene analysed and for the reference housekeeping GAPDH gene.

Gene	F-Primer	F-Primer Length	R-Primer	R-Primer Length	Accession Number
Gapdh	CTGCACCACCAACTGCTT	18	TTCTGGGTGGCAGTGATG	18	SY171038758-018/019
Hsp70	CGCGATGCCAAACTAGACAA	20	TCACCTGACAAAATGGCTGC	20	AY861684
Mt10	GCCTGCACCTTGTAACTGTAT	21	CTGTACACCCTGCTTCACAC	20	AY566248
Gst	AGTTAGAGGCCGAGCTGAA	19	TGGAAACCGTCATCATCTG	19	SY140930374-050/051
Pl III	CACCCAACAAGAAGGATGCC	20	CCTTGCCCTTTTCTTTCCCC	20	SY140930274
Pl II/IV	AAGCCCAAGTAGACGTTCCA	20	TCCGAGGTGTGATGTGTTGA	20	SY140930274

## Data Availability

Not applicable.

## Abbreviations

CT	Connective tissue
VTC	Vesicular connective tissue cells
ADGs	Adipogranular cells
17β-HSD	17β-Hydroxysteroid dehydrogenases
3β-HSD	β-Hydroxysteroid dehydrogenase/Δ^5−4^ isomerase
PL- proteins	Protamine-Like
H&E	Hematoxylin and Eosin
ASD	Androstenedione
T	Testosterone
DHEA	Dehydroepiandrosterone
n.d. MNase	Undigest Micrococcal nuclease
